# Conducting decolonizing research and practice with Australian First Nations to close the health gap

**DOI:** 10.1186/s12961-021-00773-3

**Published:** 2021-09-22

**Authors:** Pamela Laird, Anne B. Chang, John Jacky, Mary Lane, André Schultz, Roz Walker

**Affiliations:** 1 Wal-Yan Respiratory Research Centre, Telethon Kids Institute, University of Western Australia, Perth, WA Australia; 2 Department of Physiotherapy, Perth Children’s Hospital, 15 Hospital Avenue, Nedlands, WA 6009 Australia; 3 The Child Health Division Menzies School of Health Research, Darwin, NT Australia; 4 Department of Respiratory Medicine, Queensland Children’s Hospital, Brisbane, QLD Australia; 5 The Centre of Children’s Health Research, Australian Centre for Health Services Innovation, Qld University of Technology, Brisbane, QLD Australia; 6 Kulunga, Telethon Kids Institute, Broome, WA Australia; 7 Broome Regional Aboriginal Medical Service, Broome, WA Australia; 8 Department of Paediatrics, School of Medicine, University of WA, Perth, Australia; 9 Department of Respiratory and Sleep Medicine, Perth Children’s Hospital, Perth, WA Australia; 10 School of Indigenous Studies, Poche Centre for Indigenous Health, University of Western Australia, Perth, WA Australia

**Keywords:** First Nations, Participatory action research, Knowledge translation, Decolonizing methodology

## Abstract

The purpose of this paper is to highlight a perspective for decolonizing research with Australian First Nations and provide a framework for successful and sustained knowledge translation by drawing on the recent work conducted by a research group, in five remote communities in North-Western Australia. The perspective is discussed in light of national and international calls for meaningful and dedicated engagement with First Nations people in research, policy and practice, to help close the health gap between First Nations and other Australians.

## Background

Worldwide, especially in Western countries, First Nations people have poorer health outcomes and lower life expectancy compared to non-First Nations peoples [1, 2]. While the life expectancy gaps have narrowed in New Zealand, the United States and Canada, in Australia the gap has not narrowed in the last ~ 10 years [1, 3, 4]. In 2007, the Australian Government committed to achieving health equity for First Nations within 25 years [4] (i.e. by 2031), but the life expectancy gap has neither closed nor is it on target to close by 2031. Australian First Nations consists of Aboriginal and Torres Strait Islanders, but in some regions, for example in the Kimberley region of Australia, the local preferred terminology is “Aboriginal”, which is the term we will use when referring to studies in these regions. Elsewhere, we will use the term “First Nations”, which is recommended when discussing Australian Aboriginal and Torres Strait Islander people in international contexts [5].

Despite the strength and resilience of First Nations Australians, past colonial policies have contributed to cultural dislocation and cumulative multigenerational trauma for Aboriginal people [6, 7]. These historic and contemporary socioeconomic factors all contribute to the gap in health outcomes and life expectancy [8, 9]. To date, there remains widespread socioeconomic disadvantage, including but not limited to the literacy and numeracy gap, higher incarceration and unemployment rates, and lower average household income levels of Australian First Nations compared to other Australians [10]. Furthermore, until recently they had little power to influence the determinants of health or the public policy decisions affecting their health, further compounding the limited successes in closing the gap to date [11].

Indeed, the inequality in health outcomes has attracted international attention and criticism, including the United Nations [11]:

"…the failure to respect the right to self-determination and the right to full and effective participation…is alarming. [The compounded effect of these policies has contributed to the] failure to deliver on the targets in the areas of health… in the Closing the Gap strategy… I urge the Government to use this momentum to reset the relationship with the First Nations of Australia and in a collaborative manner construct a new joint pathway to the future."

Similarly, a recent Lancet editorial expressed “a feeling of déjà vu” and “utterly disappointing” following the release of the “2018 Closing the Gap report on health of Indigenous people in Australia”, where only three of the seven targets designed to narrow inequalities for Indigenous people were on track. Again, the call was for “meaningful and dedicated engagement with Aboriginal and Torres Strait Islander peoples” [12].

Explanations for the health gap between First Nations and other Australians have been widely researched and reported with a broad range of solutions suggested and tested since the 1960s. However, despite the repeated calls for solutions to target this health inequality, the gap remains. The challenge today is to heed these national and international calls and embark on a new era of community-led partnerships with First Nations at the centre [4].

In the past 2 years since the 10-year anniversary of the commencement of the “Close-the-Gap” agenda, there have been calls within Australia to rethink the existing and failed strategies. The federal government has recently recognized the failures to date and responded to both international and Australian First Nations-led calls to renew and prioritize efforts to close the life expectancy gap, by undertaking co-led initiatives [13]. In March 2019, the Australian Prime Minister entered into a Partnership Agreement on Closing the Gap. The agreement represented a commitment by the Commonwealth, states and territories, the Australian Local Government Association and the Coalition of Aboriginal and Torres Strait Islander peak organizations (i.e., nongovernmental organizations that advocate for, or lobby government, on behalf of smaller member organizations with allied interests) to work together in genuine partnerships to improve current trajectories through ensuring the expertise and involvement of Australian First Nations throughout the process, and guiding local action and local change [4]. Similarly, this approach, which includes dedicated and genuine engagement, collaborative partnerships and coproduction with First Nations stakeholders and end-users, remains the preferred strategy for First Nations research today [14, 15].

### Ensuring research is culturally secure and anti-racist

In 2013, Kerry Arabenna, a First Nations scholar, was commissioned by the Medical Journal of Australia to identify initiatives to improve the health and well-being of Australian First Nations to help close the gap [16]. Arabenna outlined the negative impact racism has had on the health of First Nations and the importance of acknowledging it:

…dubious practices, disparities in access and subtle variations in effort within health... full health equity cannot be achieved until racism…can be overcome. [16]

Similarly, one of Australia’s most recognized First Nations scholars in racism, Yin Paradies, expressed that reduction of racism can only be achieved through improving community awareness of racial prejudice, improving cultural competency among health workers and health services and redesign of health services to facilitate optimal access [17]. Such an approach is also necessary in research and is referred to as *decolonizing methodologies and practices* [18]***, ***which is strategically anti-racist. Specifically, the approach includes researcher reflexivity [19] and acknowledging and respecting Aboriginal ways of knowing, being and doing [19]. Importantly, this approach ensures that researchers recognize the unequal power inherent in the researcher–participant relationship by “disarming” themselves as the “expert”, while adopting a critically reflexive stance [20], which facilitates Aboriginal expertise and diminishes disempowerment [20]. The work undertaken should endeavour to include practices such as the establishment of a community reference group; extensive stakeholder engagement; research conduct agreements with communities; clearly delineated research outcomes communicated to the community; appointment of local First Nations co-researchers and community navigators; mechanisms to feed back results to communities; and research codesign and ongoing participatory action research (PAR) implemented throughout the research process in accordance with the National Health and Medical Research Council (NHMRC) *Ethical conduct in research with Aboriginal and Torres Strait Islander Peoples and communities: Guidelines for researchers and stakeholders* [21] and the NHMRC *Keeping research on track II* guidelines and values [22]. These guidelines emphasize six core values for all First Nations research: (i) spirit and integrity, (ii) cultural continuity, (iii) equity, (iv) reciprocity, (v) respect, (vi) responsibility. By incorporating the six values into the research design and with researchers simultaneously adopting a critically reflexive stance in relation to their own power and privilege, values and assumptions [20], best research practice, which counters racism, can follow. The implementation of such an approach facilitates two-way learning and capacity building within both medical clinics and communities. It is important to also note that the NHMRC recognizes the importance of consumer engagement in all health and medical research, irrespective of race or culture. In 2016, both the NHMRC and the Consumers Health Forum of Australia released the Statement on Consumer and Community Involvement in Health and Medical Research [23] to guide researchers in the active involvement of consumers and community members in all aspects of health and medical research.

Effectiveness of knowledge translation (KT) in Indigenous contexts is influenced by the level of integrated KT, community engagement and cultural centredness, where sustainable and effective outcomes are facilitated when such aspects are reflected in the research design [24]. There is an urgent need to incorporate high-quality translational research to facilitate improved health of First Nations that is both culturally informed and community led. This includes requiring a thorough and systematic strategy to address the problem at both community and health systems levels. The approach taken needs to consider the currently very low rates of successful translation of research into sustainable change [25–27] and understand that a collaborative, community-based approach is critical [8] and that knowledge alone is insufficient. There is currently a paucity of such data. Thus, in this paper, we aim to highlight a perspective for research with Australian First Nations and provide a framework for successful KT to improve health outcomes. The framework includes the combined use of PAR and KT science (outlined below). While the concept of a partnered approach, which incorporates Indigenous values into integrated KT research, has been previously well documented and recommended [28], the use of these methodologies has not been applied to address health inequity in First Nations contexts by addressing both health consumer and health service provider needs simultaneously. We propose that the simultaneously implemented strategies, which address the barriers and facilitators for First Nations consumers and their health providers, using combined PAR/KT methods, promotes sustained KT. We will outline a case study of successful application of this dual strategy for both clinicians and First Nations Australians, which resulted in improved health outcomes. Further, we propose this approach may have merit when implementing health systems changes and to other chronic diseases to improve health outcomes.

## Methodology

### Selection of research methodology

Using appropriate research methodologies is critical to ensure effective and sustainable KT. Only 14% of scientific discoveries translate into clinical practice, and it takes on average 17 years for successful KT to occur [29]. To compound the problem in First Nations contexts, research translation has a poor track record [30, 31]; historically, First Nations have been excluded from design and implementation and therefore, not surprisingly, fared poorly from positivistic and biomedical research agendas and their outcomes [32].

However, there are methodologies and strategies that have shown promise for sustainable change [30]. PAR and KT are two such methodologies that have been used in First Nations contexts around the world and provide an effective and culturally secure framework for conducting research in partnership with all key stakeholders to facilitate sustainable change. We suggest that KT science and PAR are two appropriate and promising methodologies in First Nation contexts [28, 30] to facilitate systems changes and improve health outcomes. We will describe how the successful combination of KT/PAR applied in four remote Aboriginal communities [33] and a regional town [34] resulted in improved health outcomes. The researchers addressed and quantified a chronic health problem by engaging with Aboriginal communities, health practitioners and key stakeholders. We will outline how the combined methods may provide a framework for real-time translational benefits and outcomes in First Nations communities.

### KT science

KT science is defined by the Canadian Institute of Health Research as “a dynamic and iterative process that includes the synthesis, dissemination, exchange and ethically sound application of knowledge to improve health, provide more effective health services and products, and strengthen the healthcare system” [28]. The primary purpose of KT is to bridge the “know–do” gap. More recently, there has been a shift towards integrated KT. Integrated KT incorporates *knowledge production* into the “know–do” gap and encompasses the complexities of health systems, including the knowledge and experiences of personnel and consumers and other relevant stakeholders [28].

Traditionally, KT science has been a research approach driven by the researchers/heath decision-makers, which aims to apply knowledge to benefit the health of the community and improve health systems. KT approaches ensure the researchers work with the “end-users” who become collaborators and partners in the research process and contribute to the creation of new knowledge [28]. Unfortunately, translation of knowledge into practice is historically often unsuccessful in First Nations contexts [30, 31] and may not produce sustainable change [31, 35]. Reasons for the failures are complex and may include failure to draw on First Nations knowledge, engage with and inform First Nations [31, 36], engage with local clinicians and health services, and/or provide healthcare to families in a culturally secure and meaningful way [37]. Participatory approaches to KT research have shown promise for increasing levels of collaboration with consumers, communities, organizations and researchers [38, 39]. PAR is a methodology that can be incorporated into KT science to facilitate effective collaborative processes.

### PAR approach

PAR is a research approach involving community members, stakeholders, organizations and researchers equally in all aspects of the research process [40]. PAR empowers and engages with communities and is widely used with First Nations as it aligns very well with ethical principles guiding the conduct of research in First Nations contexts [41–43]. The use of PAR within First Nations contexts fosters strong and connected culture, and self-determination through informed community-driven holistic responses and solutions [44–46].

PAR values the importance of genuine community ownership of knowledge and, importantly, community leadership and involvement in scale-up and implementation of research into practice [47]. PAR is designed to address complex issues through inclusion of community knowledge and resources and through creating new knowledge derived from both community and stakeholder perspectives. PAR bridges cultural differences through its collaborative processes, and by virtue of facilitating local ownership, PAR generates translation and sustainability of resources and models of care to address local issues [44, 46].

A distinction of PAR is the emphasis on community-initiated research, which recognizes the power relations between researchers and knowledge users and aims to eliminate injustices and inequities of marginalized groups [28]. PAR incorporates a holistic understanding of health and well-being, more akin to Australian First Nations’ holistic definition of health, that is, physical well-being of an individual refers to the social, emotional and cultural well-being of the whole community [48].

Aboriginal PAR (APAR) has been presented as a specifically critically reflexive and transformative Indigenous research methodology [49]. The approach is designed to increase the voice of First Nations and validate self-determination in First Nations research. One of the key distinctions of APAR from PAR, is the recognition of the sovereignty of First Nations’ knowledge. In an important sense, APAR provides a distinctive focus on decolonizing approaches, which insist on privileging First Nations voices.

### Combined KT and PAR methodologies: an innovative approach to translate First Nations research

Research strategies in First Nations contexts are more likely to be successful if particular criteria are present. These include strategies that are developed with a comprehensive and integrated approach based on identified barriers and facilitators; draw on local First Nations knowledge; culturally informed and community led; and designed to optimize healthcare provision through health system changes [30]. In short, a KT approach that encapsulates the principles of PAR has the potential to effectively undertake research that produces relatively immediate and lasting change in First Nations contexts. KT and PAR emphasize mutual respect and co-learning between partners, individuals and communities, and a balance between research and action to increase community and workforce capacity and activate systems-level change to improve health outcomes [44, 46]. Importantly, Haynes et al. demonstrated that for First Nations health research to result in real-world transformative change, it must be community-led, ensure two-way knowledge sharing, equalize power differences, integrate both biological and decolonizing social science approaches, and be guided by Indigenous strengths, knowledge and world view. It must encompass critical reflexivity (i.e., thinking about and controlling for the impact of one’s own assumptions, values and actions) to ensure one is not blinded to the influence of colonization, institutional racism and prejudice in our systems, structures and practices [50]. These characteristics align well with the combined KT and PAR approach.

Both of these methodologies can be integrated within the Consolidated Framework For Implementation Research (CFIR), a theoretical framework which outlines how the implementation process is planned, organized and scheduled [27]. The framework is a helpful construct to systematically consider the multiple and integral components necessary when planning and implementing strategies to maximize both KT and its sustainability, particularly in the health context.

The CFIR originally comprised of five domains of influence: (1) inner setting (the characteristics such as *cultural and political characteristics of the institution in which the implementation process will proceed*; (2) outer setting (e.g., community/consumers and wider governance structures); (3) individuals involved in the intervention and their characteristics (e.g., interplay between the organization and clinicians and managers, work culture, readiness for change); (4) characteristics of the intervention (e.g., stakeholder involvement in intervention development); and (5) implementation process. There are four essential activities within the fifth domain: (i) planning, (ii) engaging, (iii) executing or implementing, and (iv) reflecting and evaluating. These activities can be done in any order.

It is important to emphasize when incorporating a framework like the CFIR in First Nations contexts, *that the approach is grounded in PAR, to ensure cultural integrity and decolonizing approaches are facilitated throughout all processes*.

While the CFIR is a useful tool to evaluate and plan KT research, it is important to emphasize that the framework *is not a methodology, but rather a tool* for researchers to consider all of the important aspects when implementing and evaluating KT research. Importantly, the framework has some limitations within First Nations contexts, *and has been criticized for its underrepresentation of “patient voice” in its construct *[51]. The “inner setting” refers to factors within the organization. Patients are relegated to the “outer setting”. However, Safaeinili et al. [51] proposed creating a sixth domain called “patient needs and resources” and included them in the “inner setting”. *The sixth domain highlights the prioritization of patient needs and voice with respect to patient-centred care, which is particularly important in First Nations contexts, where decolonizing approaches are essential* [42]* and where establishing barriers and facilitators from a patient perspective is critical for health systems to consider when implementing change*. In the Australian context it is critical for clinicians and researchers to acknowledge and take into account the broader historical social, political context impacting the lives of First Nations individuals, families and communities, in thinking about the diagnosis and treatment of patients, irrespective of the disease being addressed [49, 50]. The inclusion of the sixth domain aligns with Australia’s National Safety and Quality Health Service Standards [52], which outline standards for better healthcare for First Nations consumers, and include partnerships with First Nations communities.

In order to address existing health inequities experienced by Australian First Nations, it is critical for biomedical researchers/scientists and clinicians working in the First Nations contexts to adopt culturally responsive research methodologies that value First Nations expertise and have regard for their ways of knowing, being and doing [19]. Specifically, within the PAR tradition, expert knowledge involves a commitment to decentring research “expertise” where community members’ knowledge is viewed as legitimate and expert in nature [53]. Placing equal value on First Nations knowledge and holistic conceptions of health and well-being within dominant biomedical paradigms facilitates true partnership and codesign in research. Importantly, KT and PAR methodologies require engagement with all stakeholders which includes First Nations community-controlled organizations and community members throughout all stages of the research process, from issue identification to developing research questions; to research design, data collection and analysis; and writing and dissemination and translation [44, 53].

**Fig. 1:**
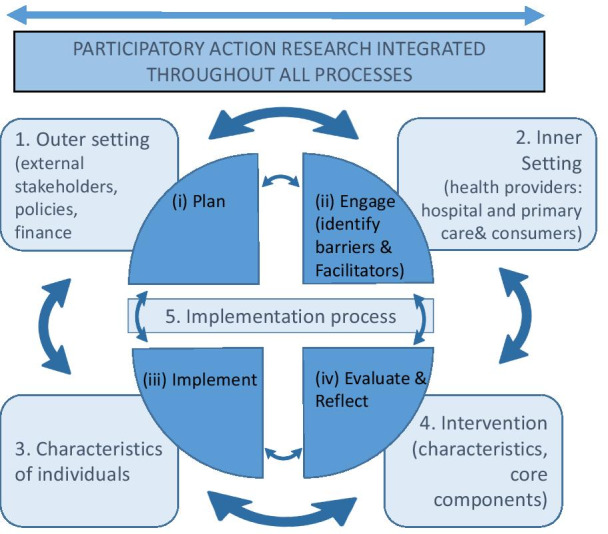
Framework of combined KT and PAR using the Consolidated Framework for Implementation Research. *Diagram adapted from Safaeinili et al. [51], Damschroder et al. [27] and Naidoo et al. [64]

Hence, research should include culturally responsive methodologies (PAR and KT) throughout all aspects of the research, including all initial planning and design being undertaken in collaboration with local First Nations research partners and health services. PAR processes incorporated in all stages of the project can reflect the ongoing needs of key stakeholders. Figure 1 depicts the combined methodologies within the CFIR.

## Framework in action: a case study

Laird et al. implemented the combined KT/PAR approach guided by the CFIR [27] when they investigated ways to improve recognition and management of chronic wet cough and protracted bacterial bronchitis in Aboriginal children by both families and primary care clinicians in a large regional town in the Kimberley region of Western Australia [34]. The research approach was directed by local leaders, that is, the Aboriginal Medical Service (AMS) board of directors and their appointed Aboriginal clinician from within the local AMS, who became the lead local researcher and coauthor (ML).

The first step in the research process was to understand the barriers and facilitators to early recognition, health-seeking and management of chronic wet cough from the community [37] and health service [54] perspective. Extensive interviews and focus groups were conducted with families and key stakeholders. The next step involved the development of strategies to address the problems. These strategies included the adaption [55], development [56, 57] and implementation of health literacy tools (flip chart [56], animated film [57]) for use by clinicians and a local health information campaign for families to improve parent/carer understanding of the importance of health seeking for chronic wet cough. This campaign was produced using codesign, local film-makers and local champions. A training programme for primary care clinicians in culturally secure assessment and management of chronic lung disease in Aboriginal children and several primary care health systems changes were implemented to improve clinicians’ ability to successfully identify and manage early lung disease. The training programme consisted of three different 1-hour in-person lectures, each a month apart, by AS (paediatric respiratory physician). The lectures included audit and feedback of clinicians’ management of children in the previous week and a quiz to test knowledge before and after the training. The training programme for clinicians was subsequently translated into an online training module for national dissemination and is endorsed by the Royal Australian College of General Practitioners and the Lung Foundation Australia [58]. System changes included facilitating follow-up of at-risk children by the same doctor to facilitate care continuity by having a 1-hour time slot for recall of patients, updating the local management guidelines to ensure they were consistent with the national guidelines, distilling the protocol into a simple flow chart displayed on the walls of consultation rooms to enable easy access and reminders for clinicians.

The study confirmed that the combined KT and PAR approach was effective [34] as it demonstrated significantly improved recognition, health-seeking and management of chronic wet cough in Aboriginal children by their families and clinicians. Specifically, the number of families seeking help for their children with chronic wet cough almost tripled following implementation of a lung health information campaign. Clinician proficiency in detection and management of chronic wet cough also improved significantly post KT as reflected in the improved parent proxy, cough-related quality of life scores in children with chronic wet cough. Furthermore, post KT, clinicians were more likely to ask patients who presented with respiratory complaints about cough (presence, quality and duration) and children with chronic wet cough were more likely to be prescribed appropriate therapy [34]. Importantly, when the researchers followed up a year later, there was evidence of ongoing translation, with some clinicians reporting they were training clinicians new to the institution in management of chronic wet cough [34].

The results of the dual strategies, which were implemented simultaneously during a 3-month period, confirmed that provision of a codesigned, community-wide, culturally secure strategy improved health seeking of families and health outcomes for Aboriginal children with chronic wet cough. The findings also strengthen the argument that improving health literacy in a culturally responsive way using codesign not only improves disease-specific knowledge [55], but also translates to improved health outcomes, increased engagement by families with primary healthcare services, and improved early detection and effective treatment of illness [59].

First Nations research leaders have long argued that to make a positive difference, health-related research needs to be done *with them not for them* – *nothing about us without us* [60, 61]. A large part of the reason for the success in translational outcomes was the extensive Aboriginal-led involvement in all aspects of the study. That is, the community, which included the two local Aboriginal-controlled health organizations, Aboriginal consumers and other community members, were consulted to identify the gaps in family and clinician knowledge about the disease. The community co-formulated solutions (based on identified barriers and facilitators) and codeveloped multiple strategies. The level of community involvement was extensive. For instance, the community identified the local champions to be the voice and face of the media campaign and the images and script of the health literacy materials. The local Aboriginal media company provided their studio and equipment to record the television and radio advertisements and teaching video. The Aboriginal radio station broadcast the commercials free of charge throughout the campaign and organized promotion and Nation-wide media interviews of disease-specific clinicians/experts to promote the health message widely. With respect to the community presentations, the families again identified the various key groups to ensure the health message was provided to the most relevant individuals and families – for example, the community requested training for the local Aboriginal day care centre staff and yarning circles (a conversational process of sharing information and stories) at all the local Aboriginal play groups. All research data, including any photographs taken, were provided back to the communities via AMS and community boards and navigator presentations, written reports and PowerPoint presentations. The community navigators and boards then disseminated the information within their communities. Co-authorship of published papers was undertaken with a locally appointed Aboriginal researcher (ML). All media, including national news television coverage, was coordinated through the local community council Elders and leaders for approval prior and was conducted with supervision of local senior Aboriginal spokespeople, who also participated in the media story. Reciprocity from the local AMS was evidenced by their directive for clinical staff to attend training in best practice management of children. Importantly, the AMS executive released an Aboriginal child health nurse (ML) and Aboriginal health workers to participate and undertake training to become co-researchers. The local Aboriginal clinical researchers provided critical support to ensure culturally secure research practices, identification and recruitment of participants, and teaching families about lung health.

The study design included:

1. Community engagement (2 years prior to project commencement). Engagement included visits to communities and their health services to yarn about lung health and for the clinician researchers (AS, PL) to share their journey to improve respiratory healthcare for Aboriginal children. Likewise, yarning allowed local community leaders to understand the health need and invite the clinical research team to begin work with the community to solve the problem.
2. Partnerships (with both AMSs). An agreement between the local AMSs and the research organization was formalized.
3. Codesign (with community leaders and councils during the engagement process). The project was adapted as required throughout the project to suit the needs of the Aboriginal families and the medical service. For example, the local Aboriginal child health nurse and Aboriginal health worker highlighted the need to adapt the process when interviewing families. The PAR design allowed the identified problem to be addressed immediately. Many families had children who had presented multiple times for their child’s chronic wet cough, but they had not received treatment. The paediatric respiratory physician researcher (AS) was available during the interviews to provide expertise in evaluating those families who wanted their child checked. The physician ensured the local clinicians had the knowledge and skills to manage the children. The refinements facilitated immediate empowerment and benefits to the community and informed implementation strategies and innovations and thereby improve the likelihood of KT [13]. The study design allowed for the adaptive changes without affecting the integrity of the methodology or without influencing the results.
4. Coproduction—for example, the Aboriginal media company and talent for the health promotion campaign were sourced locally, and the AMS medical leaders worked with AS to update the local clinical practice guidelines for management of cough in children [62] and inform the development of the training programme.
5. Co-ownership (with local AMSs, local hospital and state tertiary hospital). All materials produced as part of the study are co-branded by all stakeholders involved, and no profit can be made from their dissemination. The materials are freely available online.
6. Cultural centredness (through PAR design).
7. Capacity building—through training of local clinicians in best practice management of chronic wet cough (AS, PL), training of Aboriginal clinicians and community members as researchers (RW, PL), and training of non-Aboriginal researchers in Aboriginal culture (ML, JJ).
8. Systems change (through stakeholder engagement with AMS administrative and clinical leads.
9. End-user engagement (PAR process throughout with families and clinicians) [15].

The incorporation of the nine concepts outlined above resulted in successful and sustained KT at both a clinician and community level. To our knowledge, this is the first community-based study that addressed both community and primary care service provision barriers to the management of chronic disease in an Aboriginal context. The significant improvements in both family health seeking, lung health outcomes of children, and clinician proficiency in disease management suggest credibility in the “whole-systems” approach, that is, combined PAR/KT.

Schultz and colleagues were subsequently invited by the Kimberley AMS to expand the project to other parts of the Kimberley. Schultz is currently leading an expansion of the project to multiple sites in three Australian states. Further, the team were invited by four surrounding remote Kimberley communities to look at ways to improve lung health outcomes for children in those communities. The knowledge implemented in the original project, namely new health literacy materials, information campaign, clinician training programme and local researcher training, formed the framework to measure disease burden in the four remote communities as a whole-population screening study. Children who were identified as having disease were then able to receive immediate treatment at their local clinic, where the clinicians had received training in best practice management of the targeted condition (chronic wet cough).

Importantly, 76% of families of children identified with chronic wet cough attended their local clinic and received appropriate antibiotic management. Further, all of those children reportedly completed the treatment, were followed up locally and their symptoms resolved. This suggests that informed parents seek help and adhere to the required management, highlighting that “knowledge is power” and the effectiveness of culturally secure health information for families. The community-led initiative is further evidence that PAR design can positively impact health outcomes for First Nations. It showcases how some studies can be designed to provide real-time translational change without compromising the rigor of scientific research.

The combined PAR/KT approach ensured that local Aboriginal community members and clinic staff were trained in research skills necessary for the project prior to commencement. Key community navigators directed the project at their community to ensure the cultural integrity of all processes. Pairs of research teams were formed for each community. Each pair included one Aboriginal researcher and one lead clinician-researcher (one male and one female in each team) to undertake the research process with a mix of male and females and language group representatives to ensure respectful engagement according to local cultural requirements. The project successfully recruited 94% of the possible whole population of children under the age of 7 years. The high recruitment rate is unusually successful for research and confirms the acceptability of the approach by the community and the value of employing Aboriginal researchers. Further testament to the approach was confirmed 12 months later when the group were invited to continue working with the communities to expand to all children aged to 18 years and to include ear health screening. The expansion is currently underway, where more than 90% of children in each community have been enrolled to date.

### The case for a combined KT/PAR approach

While there is rationale for exclusive use of either a KT or PAR approach, the limitations for both approaches have been outlined. In essence, the case presented above, which incorporated a combination of both methodologies, has encapsulated the six core values of First Nations research in Australia and translated to real-time meaningful health outcomes for First Nations children. KT alone would not have been sufficient to capture the voice and needs of First Nations families, nor would the project have enjoyed the successful outcomes without the community codesign. Similarly, the PAR approach greatly benefitted by adopting the KT systematic approach to include all core components necessary to implement health systems changes within complex systems. For instance, there was a key need to train clinicians in best practice guidelines to identify and manage early disease. There was little community and clinical awareness of the need to manage the early signs of disease in children. The systematic implementation of evidence-based knowledge, which is a hallmark of KT science, ensured the successful implementation of widespread practice change. Lastly, the CFIR embedded within the combined methodology facilitated systematic and measurable steps with clear identification of core components integral to ensure successful KT with a sustained outcome.

## Conclusion

Culture-centred approaches incorporating high levels of community engagement improve the likelihood of research translation and sustainability [15, 50], thereby making a case that the use of integrated PAR/KT, which addresses both health consumer and health provider needs, is an effective, culturally responsive method. In Australia, there is now strong policy contexts at the state, territory and national levels [63], with genuine commitment to close the First Nations health gap through both health and research initiatives and a refreshed “Close the Gap” strategy [4]. This confirms and emphasizes the need for partnerships between health researchers and clinicians, First Nations consumers, communities and stakeholders. In particular, it is important that research employs a community-led PAR approach, which privileges and incorporates the voice, knowledge and expertise of First Nations. The evidence provided in this paper provides a compelling case to incorporate KT and PAR within research translation approaches, to centre First Nations voices to bring about real-time changes across the health system to improve health outcomes.

## Data Availability

Not applicable.

## Abbreviations

NHMRC: National Health and Medical Research Council
PAR: Participatory action research
KT: Knowledge translation
APAR: Aboriginal participatory action research
CFIR: Consolidated Framework for Implementation Research
AMS: Aboriginal Medical Service
